# Supplementary material for: Race, employment, and the pandemic: An exploration of covariate explanations of COVID-19 case fatality rate variance
Source: PLoS One. 2023 Feb 2;18(2):e0274470. doi: 10.1371/journal.pone.0274470 (PMC9894486; doi:10.1371/journal.pone.0274470)
Supplement: S1 File — (PDF) [file pone.0274470.s002.pdf]

## Supporting information

**Fig 9.** Relationship between current unemployment and minority population proportion.

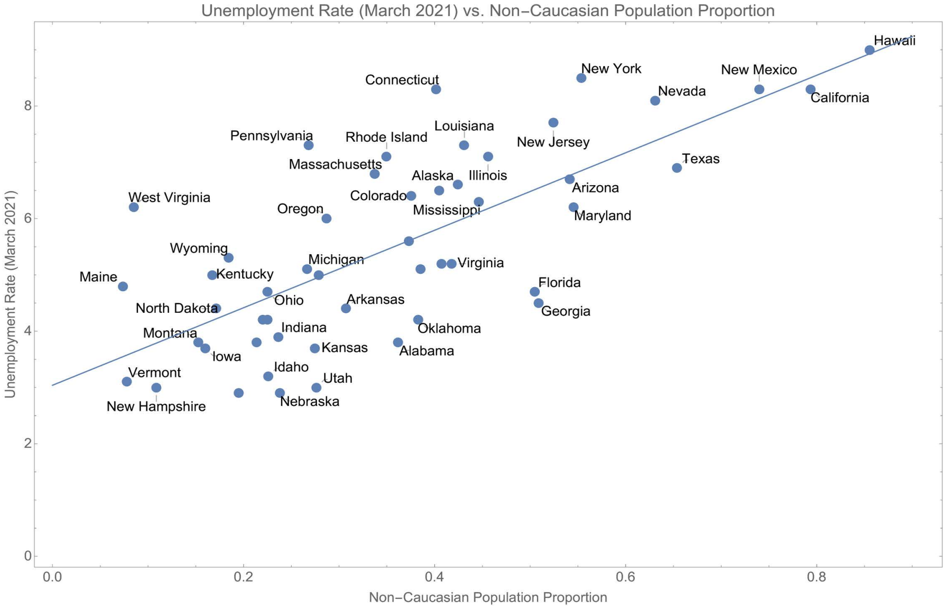


| Mon-Yr | Unemployment vs. CFR Correlation p-Value |
|---|---|
| May-20 | 0.0056 |
| Jun-20 | 0.00056 |
| Jul-20 | $9.312 \times 10^{-6}$ |
| Aug-20 | 0.0018 |
| Sep-20 | 0.0032 |
| Oct-20 | 0.0262 |
| Nov-20 | 0.0132 |
| Dec-20 | 0.00002 |
| Jan-21 | 0.00002 |
| Feb-21 | 0.00012 |
| Mar-21 | 0.00004 |

**Table 4.** The correlation between CFR and unemployment rate, month-by-month from May 2020 - March 2021.

| Statistic | Value |
|---|---|
| Min | 2.9 |
| $25^{th}$ Percentile | 4.2 |
| Mean | 5.48 |
| Median | 5.15 |
| $75^{th}$ Percentile | 6.8 |
| Max | 9. |
| Standard Deviation | 1.71 |
| Kurtosis | 2.01 |
| Skew | 0.28 |

| Statistic | Value |
|---|---|
| Min | 2.4 |
| $25^{th}$ Percentile | 3.2 |
| Mean | 4.22 |
| Median | 3.6 |
| $75^{th}$ Percentile | 4.8 |
| Max | 8.3 |
| Standard Deviation | 1.61 |
| Kurtosis | 3.35 |
| Skew | 1.23 |

**Table 5.** (Left) Descriptive statistics for current unemployment data used (percent). (Right) Descriptive statistics for projected unemployment using Eq. (3) (percent). Additional unemployment data is available in a data archive provided with this paper.

| **Statistic** | **Value** |
|---|---|
| Min | 30.5 |
| $25^{\text{th}}$ Percentile | 36.8 |
| Mean | 38.2 |
| Median | 38.25 |
| $75^{\text{th}}$ Percentile | 39.4 |
| Max | 44.3 |
| Standard Deviation | 2.39 |
| Kurtosis | 4.53 |
| Skew | -0.17 |

| Statistic | Value |
|---|---|
| Min | 1.11 |
| $25^{\text{th}}$ Percentile | 43.11 |
| Mean | 178.01 |
| Median | 93.00 |
| $75^{\text{th}}$ Percentile | 202.33 |
| Max | 1065.57 |
| Standard Deviation | 226.33 |
| Kurtosis | 8.20 |
| Skew | 2.30 |

**Table 6.** (Left) Descriptive statistics for median age data (years). (Right) Descriptive statistics for population density (people per square mile). Additional demographic information is provided in the data archive provided with this paper.

| **Statistic** | **Value** |
|---|---|
| Min | 0.005 |
| $25^{\text{th}}$ Percentile | 0.033 |
| Mean | 0.11 |
| Median | 0.07 |
| $75^{\text{th}}$ Percentile | 0.15 |
| Max | 0.38 |
| Standard Deviation | 0.095 |
| Kurtosis | 3.47 |
| Skew | 1.14 |

**Table 7.** Descriptive statistics for African American proportions per state (proportion of population). Additional demographic information is provided in the data archive provided with this paper.

| **Statistic** | **Value** |
|---|---|
| Min | 0.005 |
| $25^{\text{th}}$ Percentile | 0.015 |
| Mean | 0.017 |
| Median | 0.017 |
| $75^{\text{th}}$ Percentile | 0.02 |
| Max | 0.028 |
| Standard Deviation | 0.005 |
| Kurtosis | 3.41 |
| Skew | 0.18 |

| **Statistic** | **Value** |
|---|---|
| Min | 0.005 |
| $25^{\text{th}}$ Percentile | 0.012 |
| Mean | 0.015 |
| Median | 0.014 |
| $75^{\text{th}}$ Percentile | 0.018 |
| Max | 0.026 |
| Standard Deviation | 0.005 |
| Kurtosis | 2.85 |
| Skew | 0.11 |

**Table 8.** (Left) Descriptive statistics for instantaneous case fatality rates (proportions). (Right) Descriptive statistics for asymptotic case fatality rates computed with Eq. (1).
