# Supplementary material for: Race, employment, and the pandemic: An exploration of covariate explanations of COVID-19 case fatality rate variance
Source: PLoS One. 2023 Feb 2;18(2):e0274470. doi: 10.1371/journal.pone.0274470 (PMC9894486; doi:10.1371/journal.pone.0274470)
Supplement: Attachment — Submitted filename: main.pdf [file pone.0274470.s003.pdf]

Response to Reviewers Comments on *Race, employment, and COVID-19: an exploration of covariate explanations of COVID-19 case fatality rate variance (PONE-D-22-10078)*

Christopher Griffin Ray Block Jr. Justin D. Silverman Jason Croad
Robert P. Lennon

July 29, 2022

We thank the reviewers and editors for the time they took to read and comment on our manuscript. We have made several modifications to the paper based on the reviewer's suggestions. Areas of the manuscript where large changes have been made are highlighted in blue for easy comparison.

In summary we have made the following revisions:

1. Added additional background material to the introduction and conclusions.
2. Added a thorough discussion of all symbols/variables used and a discussion on the linearization technique used to develop the model (with references).
3. Included a justification for the use of r^2 values as goodness of fit measures.
4. Provided additional data tables and a data archive with a Mathematica notebook that can be used to reconstruct all models and plots used in this paper. We also used this to correct minor typographical errors in some reported statistics. None of these errors changed the interpretation of the results. The Mathematica notebook and paper are internally consistent.

1 Reviewer #1

Comment 1.1. *This is an interesting study and the author addressed everything perfectly. In this study, the author explained the models and showed the results with online data.*

Response: Thank you very much.

Comment 1.2. *Overall, this is interesting but may be a little more introduction and discussion would be more helpful.*

Response: We appreciate the opportunity to expand upon the ideas of race and class-based inequality, and we have made substantial revisions to the "Introduction" and "Discussion" sections of the manuscript. We hope that the revised manuscript does a better job of communicating the substantive importance of this research while also putting the empirical findings

into richer context.

We added the following sentences—which further clarify the need for race/class analyses—to the second-to-last paragraph of the introduction (see page 2).

> We therefore join the growing body of "disparities" scholarship by taking race/class associations seriously, particularly as they pertain to the coronavirus pandemic. In many ways, COVID-19 has exacerbated pre-existing social inequalities, deepening the divisions between rich and poor, White and non-White, etc. ... Focusing on unemployment as a key economic indicator, we investigate the role that race and class play in explaining variations in COVID-19 fatality rates.

Please note that this edit also includes several additional citations (beyond the ones currently listed in the original manuscript):

- Block Jr, Ray, Arthur Berg, Robert P. Lennon, Erin L. Miller, and Marcella Nunez-Smith. "African American adherence to COVID-19 public health recommendations." *HLRP: Health Literacy Research and Practice* 4, no. 3 (2020): e166-e170.
- Block Jr, Ray. "Race, the Health Literacy Gap, and COVID-19: Reflections from a Social Scientist Studying 'Pandemic Politics'." *HLRP: Health Literacy Research and Practice* 5, no. 2 (2021): e121-e123.

For the discussion section of the revised manuscript (the final paragraph, which begins at the bottom of page 9, continued onto page 10), we added the following text to emphasize the broader implications of our research findings, both to COVID-19 policy and US race relations more generally.

> More generally, our research speaks to the need for careful race/class analyses, for this need becomes especially apparent if one considers the pandemic's disproportionately adverse impact on both "health-" and "wealth-" related outcomes in African American communities ... As we note in the introduction, unequal outcomes across all aspects of the COVID-19 pandemic have highlighted systemic racial injustice in our society, and are appropriately driving calls for systemic change. The present paper offers data-driven support for such change... Our work underscores the idea that the disparate impacts of COVID-19 must be both understood and addressed within an explicit framework of how race and class structure myriad arenas of American public life.

This new text includes references to the following additional citations:

- Abrams, Brooks and Ray Block Jr. "Black People Are in No Rush to Re-Open Economy Despite Financial Hits." *theGRIO* (June 11, 2020).
- Andrasfay, Theresa, and Noreen Goldman. "Reductions in 2020 US life expectancy due to COVID-19 and the disproportionate impact on the Black and Latino populations." *Proceedings of the National Academy of Sciences* 118, no. 5 (2021): e2014746118.

- Brand, Jennie E. "The far-reaching impact of job loss and unemployment." *Annual Review of Sociology* 41 (2015): 359.
- Mügge, Daniel. "Studying Macroeconomic Indicators as Powerful Ideas." In *Ideas, Political Power, and Public Policy*, pp. 108-125. Routledge, 2018.

2 Reviewer #2

Comment 2.1. *Please define terms. ex: κ, δ [etc.]*

Response: Thank you for your suggestion. We have added the following text to the manuscript:

> Here, κ and δ adjust the case fatality ratio to account for the initial condition (current value) and α and β are rate constants. The terms C_∞ and D_∞ are the limiting values of case load and the deceased population respectively.

Also:

> Here, $A = -\kappa/D_\infty$, $B = -\delta/D_\infty$ and $r_\infty = 1/K$ is the long-run case fatality rate.

Comment 2.2. *Please explain why you assume "linearized forms of solutions". May be useful for readers who aren't familiar with this model.*

Response: Thank you for your suggestion. We have added the following text:

> SIRD models are inherently non-linear, usually having no closed form solution. Using a Taylor approximation, we can linearize the right-hand-side of the differential equations [38]. This allows us to approximate a closed form solution. Assuming a constant population size then linearizing about the current state of the compartmented epidemic model, the linearized solutions have form:

Comment 2.3. *Adj.* $r^2 \approx 50 - 60\%$ *is not that good. Please justify or use other criteria too if possible.*

Response: Thank you for your comment. We have added a literature review (in the Materials and Methods section) on the us of r^2 and justify our use of this as a goodness of fit measure.

> We use Adjusted-r^2 (rather than p-values) as one of our primary measures of goodness of fit. The goal of r^2 in this context is to assess whether specific predictors or explanatory variables have a significant effect on the dependent variable. Many of our Adjusted-r^2 values fall between 0.5 and 0.6. Ozili [40] discusses acceptable r^2 values concluding that in social science, with its erratic human-behavior data, r^2 values between 0.1 and 0.5 are considered adequate provided some/most of the explanatory variables are statistically significant, r^2 between 0.5 and 0.99 are considered quite good. This is reflected in clinical data, where studies with $0.2 < r^2 < 0.4$ have been accepted [41]. Even traditional regression analysis would consider an $0.5 < r^2 < 0.7$ to have a moderate effect size [42] and Henseler et al. [43] proposed general guidance that r^2 of 0.5 was "moderate" (compared to r^2 of 0.75, which was considered substantial and r^2 of 0.25, which was considered weak).

Comment 2.4. *Since data are available, provide a reproducible code if possible. Report descriptive statistics of data for the variables you used.*

Response: Thank you for this suggestion. We have added tables in the supplementary information that contain descriptive statistics for all covariates used in regressions. This is noted in the text. We have also provided a data archive with all data used in this paper and an executable Mathematica notebook that will reconstruct every model used (and some others that are not discussed).
